# Regeneration and Regrowth Potentials of Digit Tips in Amphibians and Mammals

**DOI:** 10.1155/2017/5312951

**Published:** 2017-04-10

**Authors:** Yohan Choi, Fanwei Meng, Charles S. Cox, Kevin P. Lally, Johnny Huard, Yong Li

**Affiliations:** ^1^Department of Pediatric Surgery, University of Texas McGovern Medical School, Houston, TX 77030, USA; ^2^Center for Stem Cell and Regenerative Medicine, The Brown Foundation Institute of Molecular Medicine for the Prevention of Human Diseases (IMM), The University of Texas Health Science Center at Houston (UT Health), Houston, TX 77030, USA; ^3^Department of Orthopaedic Surgery, University of Texas McGovern Medical School, Houston, TX 77030, USA; ^4^Center for Tissue Engineering and Aging Research, The IMM, The University of Texas Health Science Center at Houston (UT Health), Houston, TX 77030, USA; ^5^Center for Regenerative Sports Medicine, Steadman Philippon Research Institute, Vail, CO, USA

## Abstract

Tissue regeneration and repair have received much attention in the medical field over the years. The study of amphibians, such as newts and salamanders, has uncovered many of the processes that occur in these animals during full-limb/digit regeneration, a process that is highly limited in mammals. Understanding these processes in amphibians could shed light on how to develop and improve this process in mammals. Amputation injuries in mammals usually result in the formation of scar tissue with limited regrowth of the limb/digit; however, it has been observed that the very tips of digits (fingers and toes) can partially regrow in humans and mice under certain conditions. This review will summarize and compare the processes involved in salamander limb regeneration, mammalian wound healing, and digit regeneration in mice and humans.

## 1. Introduction

Mammalian fingertips and toes can partially regrow under certain conditions; however, regeneration is greatly limited compared to urodele amphibians such as newts and salamanders that can completely regrow an amputated limb [1–3]. The question is why there is such a difference between the regenerative potentials of mammals and amphibians. Embryonic, neonatal, and adult mice can regenerate digit tips if the amputation is midway through the third phalanx [4–6]; however, if the amputation occurs proximal to the midway point of the third phalanx in mice, regeneration of the digit tip does not typically occur [7, 8]. Similarly, young patients have also been documented to regrow the tips of amputated fingers if treated conservatively [9–11]. Although adults and even elderly individuals have potentially regenerated amputated digit tips, the regenerative process may not be as efficient as it is in younger patients and usually results in fibrous scars in adults. The regeneration process of the digit following injury may be related to the age of the host, with decreased restoration in adults compared to fetal or neonatal mammals [8, 10–12]. Injured adult mammalian tissues are usually replaced with fibrotic scar tissue, whereas scarless healing typically occurs in fetal wound healing which results in complete tissue recovery [13–15]. Stem cell activation and scarless wound healing are considered to be essential requisites for quality tissue regeneration [16–18]; however, for some regenerative processes a dedifferentiation process, but not stem cell activation, is required [19]. This review will summarize the literature in the context of amputated digit regeneration and beyond.

## 2. Salamander Limb Regeneration

Studies of axolotl regeneration are ongoing in order to understand the differences between regenerating and nonregenerating wounds. Full-limb regeneration in adult urodele amphibians occurs in several overlapping stages including wound healing, dedifferentiation, and redevelopment, which is similar to natural embryonic limb development [20]. The first phase in wound healing involves the contraction of blood vessels and growth of the injured epidermis to cover the remaining limb stump. Blastema cells then accumulate underneath the healed epidermis, which forms a thickened structure at its apex, called the apical epithelial cap (AEC) [21, 22]. The proliferating blastema cells of newts consist of dedifferentiated cells derived from muscle, bone, skin, and other tissues, which serve as progenitors for regenerating the new limb. However, in axolotls stem cell activation in the form of satellite cells may also play a role in blastema formation [19]. Regeneration occurs by completely different mechanisms between these two different salamander species; thus care must be taken when interpreting results between newts and axolotls. Blastema and AEC formation are dependent on the activation of some unknown signals and several known signals such as ionic fluxes, nitric oxides, MARCKs protein, and trophic factors (e.g., the FGF, TGF, and BMP families) [22, 23] in the wound that consequently promote the formation of the blastema and the AEC. The growth and differentiation phase of the regenerative process includes many features recapitulating embryonic limb development but does exhibit some differences compared to development de novo, for example, the size of the new limb, connection to the existing adult limb, and a nerve requirement [24].

### 2.1. Blastema Formation

The blastema is a group of cells originating from the limb tissue localized at the amputation site. The essential role of the blastema in limb regeneration has been investigated by Stocum and Cameron [25]. The cellular origin of blastemal cells, mechanisms of cellular release from mature tissue, dedifferentiation, accumulation of cells, blastema growth, and tissue patterning have all been the focus of extensive investigations.

#### 2.1.1. Dedifferentiation

**Figure 1:**
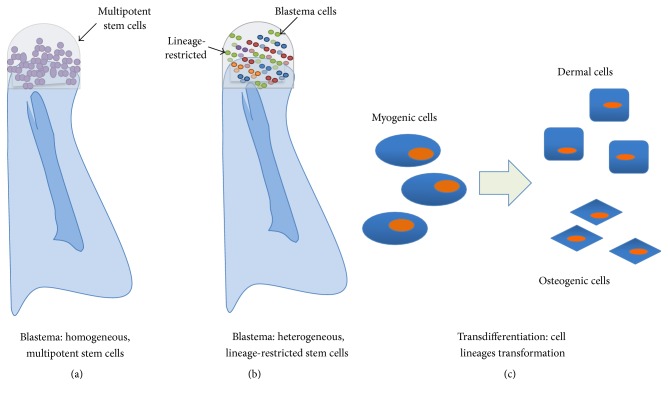
The blastema is a group of cells originating from the limb tissue local to the amputation site. (a) It was originally speculated that the blastema is a homogeneous structure of multipotent cells (purple dots), which would then form all the structures of the amputated digit tip or limb. (b) However, recent studies in the regeneration of both axolotl limb and zebrafish fin have demonstrated that the blastema cells are a heterogeneous assortment of lineage-restricted, unipotent progenitor cells (colorful dots). (c) Cell transdifferentiation might also play a role in the formation of blastema cells. Dermis and skeletal tissue, both of lateral plate mesodermal origin, have been shown to transdifferentiate. The blue area in (a, b) represents the remaining tissues following digit amputation.

It was previously speculated that the blastema was comprised of a homogeneous population of multipotent cells (Figure 1(a)) that eventually form all the structures of the amputated digit tip or limb [26, 27]. An earlier study introduced fluorescent dextran-labeled myotubes into a regenerating limb stump and found the dye in the regenerated muscles and, in limited cases, the cartilage [28], suggesting the possibility that myofibers were capable of dedifferentiating into stem/progenitor cells and contributed to tissue regeneration. However, the possibility that the cells fused [29, 30] or that the dye leaked from the muscle into the cartilage cells when the myofibers dedifferentiated into single cells cannot be ruled out. Studies in both axolotl limb and zebrafish fin regeneration, using GFP- or transposon-based clonal analysis, have demonstrated that the cells are lineage-restricted, which suggests that the blastema is a heterogeneous assortment of lineage-restricted progenitor cells [31, 32] (Figure 1(b)). The cells may undergo dedifferentiation, but not completely to a multipotent state, as cell fates are limited to their developmental origin [33]. The dedifferentiation of Schwann cell precursors also releases paracrine factor to affect mammalian digit regenerations [17]. Muscle cells from presomitic mesoderm, Schwann cells from the neural fold, and epidermis from the lateral ectoderm are all derived from the same germ layer prior to maturity. In the past decades, many studies have presented evidence favoring the view that dedifferentiation with cell lineage switching occurs during newt limb regeneration, especially when the normal regenerative process is challenged (e.g., by irradiation or loss of a particular tissue). However, other studies in which axolotls were used suggested that stem cells are primarily involved (at least for muscle regeneration) and that lineage switching does not occur. More recently, a published study showed that, during limb regeneration, muscles were regenerated by completely different mechanisms in these two salamander species: (1) dedifferentiation, proliferation, and redifferentiation in newts and (2) satellite cells in axolotls [19, 33, 34]. Therefore, lineage switching may occur in newts under certain conditions, while this does not appear to occur in axolotls.

#### 2.1.2. Resident Stem Cells

It is strongly believed that the cells in the blastema originate from dedifferentiated local tissue at the amputation site; however, adult stem cells (e.g., muscle satellite cells and possibly also the periosteum and dermis) [35–39] also contribute to the formation of the blastema, though the number of these endogenous cells may be insufficient to facilitate regeneration on their own. It has been shown that resident tissue stem/progenitor cells, rather than hematopoietic cells, contribute to the regeneration of amputated mouse digit tips [40, 41]. Adult stem cells in the nail bed are also thought to be involved in the regrowth of the amputated digit tip [42, 43].

#### 2.1.3. Transdifferentiation

Transdifferentiation is a term typically used to describe a change in cell type from one mature cell type to another, also known as lineage reprograming [44]. Cellular transdifferentiation may also play a role in the tissue regeneration process. Dermis and skeletal tissue, both of lateral plate mesodermal origin, have been shown to transdifferentiate (Figure 1(c)) [45–47]. Transdifferentiation also appears to occur in lens and retina regeneration, where the pigmented epithelial cells dedifferentiate and then form lens epithelial cells or retinal neurons, respectively [1]. The term “transdifferentiation” has been used in the literature to refer to different, but related, processes, depending on whether the cells undergo a dedifferentiation process. Some use the general term “metaplasia” instead, which is independent of the mechanism used by cells to convert to a different cell type.

#### 2.1.4. Extracellular Matrix (ECM) Involvement

Blastema cells may originate from host cells that are released from the tissue following injury-induced ECM breakdown. As cells are converted from a quiescent, fully differentiated state into a dedifferentiated state in the local surrounding matrix, many cellular changes occur. Actin cytoskeletal rearrangement, integrin disconnection from the matrix, and loss of cell polarity may induce the cells to suppress differentiation genes and upregulate genetic programs that allow the cells to reenter the cell cycle and consequently reacquire a state of “stemness” [25]. Alternatively, factors embedded in the ECM, such as cytokines, growth factors, and matrix cryptic peptides, are released upon the breakdown of the ECM and activate signaling pathways that trigger cellular dedifferentiation [48]. In addition to the stem/progenitor cell population, neural input/regrowth is also very important for the formation of the blastema [24]. In the absence of axons, the AEC forms but is not maintained, and the blastema never develops. If the nerves are removed after the blastema has formed, the limb will regenerate, but only to a limited extent, due to limited cell proliferation in the blastema. It is thought that the newly regenerated nerves stimulate the AEC to produce anterior gradient protein (AGP), which promotes the regeneration of denervated limbs [24, 49].

### 2.2. Blastema and the AEC

The AEC releases directional guidance signals to the blastema, allowing it to grow in the proper orientation. Two of the factors involved include transforming growth factor beta 1 (TGF-*β*1) and fibronectin, which are upregulated during blastema formation. Inhibition of TGF-*β*1 via SB-431542 decreases fibronectin expression and prevents blastema formation [50]. Conversely, signals from the blastema, such as the release of insulin-like growth factor (IGF), also trigger a response from the AEC [51]. Additionally, the cells in the blastema must proliferate to create enough progenitor cells to regrow the missing limb. The formation of blastema cells that accumulate under the AEC is not a recapitulation of embryonic limb development; it is a process that sets the urodeles apart from other tetrapod taxa [52, 53]. Various factors have been reported that promote blastema cell proliferation, including fibroblast growth factor- (FGF-) 1, 2, 8, and 10, transferrin, neuregulin, substance P, and AGP [54–57]. Although blastema cells proliferate rapidly, the cells of the AEC appear to be nonproliferative [49, 58], although migrating cells from the AEC do proliferate at later times [52, 53].

Patterning of the blastema cells into functional mature tissues has also been studied using various grafting experiments, which has demonstrated that the signals involved in reforming the tissues originate from the blastema [59]. A review by Tamura et al. describes several grafting experiments that demonstrate the positional memory of the blastema [60]. For example, a wrist-level blastema grafted to a more proximal stump did not grow until regeneration reached the wrist level, and the grafted blastema then grew into a supernumerary autopod (hand) [26]. Moreover, positional identity was found to be cell type-specific, such that cartilage-derived blastema, but not Schwann cell-derived cells, retained their positional identity [31, 61]. Some other experiments have demonstrated that blastemal cells have positional memory [31]. It is thought that fibroblastic cells may perform a similar function of maintaining positional identity in digit tip regeneration, because connective tissue fibroblasts from the terminal phalanx differ from those of the subterminal phalanx [62].

### 2.3. The AEC and AER

Limb regeneration partially recapitulates portions of embryonic limb development where the early developing embryo forms limb buds. The formation of the AEC is suggested to be a recapitulation of the apical ectodermal ridge (AER), a thickened epithelium at the distal end of the limb bud that functions as a signaling pathway to induce cell proliferation and maintains the mesenchymal cells in an undifferentiated state. The limb bud stops proliferating and begins to differentiate as the AER disappears [63]. The AER and AEC are considered to be functionally equivalent, with similar gene expression patterns, including the expression of* FGF-8* and* Sp-9* [64]. Proximal-distal patterning in the developing limbs is regulated by poorly understood interactions between FGFs secreted by the AER and Sonic hedgehog secreted from a posterior section of the limb bud, which in turn regulate the* Hox* genes [65, 66]. Retinoic acid regulates the* Meis homeobox* genes, which also affect proximal-distal patterning during both development and limb regeneration [67].

### 2.4. ECM Remodeling and MMP Activity

The ECM supports the architecture of the tissue during tissue regeneration. The activities of acid hydrolases and matrix metalloproteinases (MMPs), though traditionally known to play a role in mediating ECM turnover, have recently been demonstrated to actively participate in the regeneration process [68]. Regeneration failed in newts when amputated limbs were treated with the MMP inhibitor GM6001, demonstrating the essential involvement of MMPs in the regeneration process [69]. A comparison of normal, regenerating axolotls with regeneration-deficient short-toed axolotls revealed lower levels of MMP-8, MMP-9, and MMP-10 after amputation in the nonregenerating mutants, further highlighting the importance of MMPs in the regeneration process [70]. On the other hand, the participation of tissue inhibitors of metalloproteinases (TIMPs) is required to prevent excessive tissue hydrolysis and degradation induced by MMPs [71] such that dissociated cells at the amputation site begin to dedifferentiate into a more plastic stem cell phenotype [70].

Apart from the MMP activation in the early regenerative process, a transitional ECM develops that includes tenascin C, hyaluronic acid, and fibronectin, while the presence of collagens is reduced. Data suggest that tenascin C and hyaluronic acid can play instructive roles in the regenerative process [72, 73].

## 3. Mammalian Wound Healing

Many theories have been proposed to explain why successful regeneration occurs in urodele amphibians but not in mammals. First, the immune system has been shown to play a major role in the regeneration process of amputated limbs in newts [66, 74]. In mammals, fetal wounds can regenerate because they have an immature immune system; however, in adults, clearing pathogens appears to be evolutionarily favored compared to retaining the ability to regenerate a limb or digit [75]. Second, amphibians have retained limb regeneration-specific genes not found in mammals, which allow their cells to dedifferentiate [25]. A related theory is that mammals have evolved tumor suppression genes that inhibit regeneration [76]. The* Ink4a *locus is present in mammals but not amphibians; this region encodes the tumor suppression genes* p16ink4a* and Alternative Reading Frame* (ARF)*. Inactivation of both tumor suppressors retinoblastoma* (Rb)* and* ARF* allows terminally differentiated mammalian muscle cells to dedifferentiate [76]. An extension of this theory is that differentiated mammalian tissues can regenerate if the cells are induced to reenter the cell cycle, which occurs in the Murphy Roths Large (MRL) mouse and the p21-deficient mouse described below. Third, bioelectric signaling (e.g., membrane voltage polarity, ionic channels) may also play a role in the tissues' regeneration potential. Nonregenerating wounds display a positive polarity throughout the healing process, whereas in regenerating animals the polarity is initially positive but then quickly changes to negative polarity with the peak voltage occurring at the time of maximum cellular proliferation [77].

**Figure 2:**
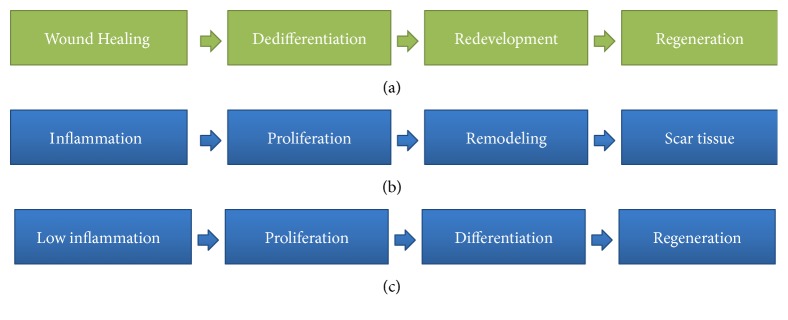
Amphibian regeneration versus (a) attempted regeneration in mammals (b and c).

Wound healing is a complex process that is not yet fully understood. Mammalian wound healing of the skin and all organ systems has traditionally been divided into three major stages: inflammation, proliferation, and tissue remodeling [78]. Attempts have been made to correlate these three stages of mammalian wound healing with the three stages of amphibian regeneration (wound healing, dedifferentiation, and redevelopment) [79]. The phases of the regeneration processes in amphibians and mammals are summarized in Figure 2.

### 3.1. Inflammation

Immediately after injury, the body responds by stopping bleeding, which involves endothelial cell vasoconstriction and the activation of coagulation pathways. Platelets coagulate to form a fibrin clot comprised of collagen, fibronectin, and thrombin, while simultaneously releasing trophic factors and inflammation-associated cytokines. Neutrophils are the initial inflammatory cells that are recruited to the wound site. They release proteases and create reactive oxygen radicals to kill invading microbes and digest damaged tissue [80]. Monocytes are next recruited to the wound site and are converted into macrophages, while the neutrophils begin to undergo apoptosis. Macrophages remove bacteria, cellular debris, and dead neutrophils via phagocytosis and release signals that recruit more macrophages and fibroblasts to the wound site. It is unclear whether macrophages and/or neutrophils are absolutely required for wound healing, because a mutant mouse model that is deficient in macrophages and functional neutrophils is still capable of healing small wounds without creating an inflammatory response and heals without scar tissue formation [81].

### 3.2. Proliferation

The proliferation stage begins approximately 4 days after injury and lasts for 10 days or more. During this period, epithelialization occurs via the expansion of skin keratinocytes. Some of inflammatory cytokines [interleukin-1 (IL-1) and tumor necrosis factor-*α* (TNF-*α*)] stimulate fibroblasts to synthesize and secrete keratinocyte growth factor-1 (KGF-1), KGF-2, and IL-6, which signal the keratinocytes to migrate and proliferate. Regulators of reepithelialization also include hepatocyte growth factor (HGF), FGFs, and epidermal growth factors (EGFs) released from injured tissues, which can stimulate receptor tyrosine kinases [82]. In contrast, TGF*β* inhibits keratinocyte proliferation, and mice with mutations disrupting the TGF*β* pathway have been observed to display faster wound healing [83, 84]. The provisional fibrin and fibronectin matrix formed during the inflammatory stage is reinforced by proteoglycans and other proteins synthesized by fibroblasts, which is then replaced by a stronger, more organized matrix composed of types I and III collagens. T lymphocytes migrate into the wound site after macrophage and fibroblast infiltration and are thought to influence the proliferative phase of wound healing [85]. Angiogenesis is stimulated by vascular endothelial growth factor A (VEGFA) and FGF-2, which stimulate endothelial cells to proliferate and form capillaries. Fibroblasts then transform into myofibroblasts to close the wound as a result of TGF-*β*1 and PDGF signaling [86].

### 3.3. Tissue Remodeling

The third phase of wound healing is tissue remodeling, which begins about a week after injury but can last for months or years after injury [87]. The remaining cells either migrate out of the wound or undergo apoptosis, leaving a scar consisting of mostly collagen and other ECM proteins and very few cells. During remodeling, type III collagen in the matrix is remodeled to the stronger type I collagen via MMPs, reducing the total type III collagen from 30% to approximately 10% [88]. Scar formation is the result of excess, unorganized collagen deposition [85] and is thought to be a mechanism to prevent the entry of microorganisms and to quickly provide mechanical support. Scars on the skin do not regrow hair follicles or sweat glands and are more sensitive to UV radiation [89].

After injury, basement membrane formation differs in the wound healing response between mammals and amphibians. In normal skin (both in mammals and in amphibians) the basement membrane lies between the epidermis and dermis and is comprised of collagen fibers, laminin, and other components. In mammals, a new basement membrane is formed between the new epidermis and dermis, which is then maintained during the wound healing process. This supports tissue integrity at the expense of scar formation. However, the basement membrane does not form during healing and only appears after regeneration is complete in amphibians [90]. If the basement membrane is induced in amphibians before regeneration is complete, scar formation occurs and regeneration ceases [91]. The basement membrane, however, may also play a beneficial role, as wound healing is impaired in mice lacking the basement membrane component nidogen 1 [92]. Nidogens 1 and 2 are basement membrane proteins that interact with laminin, collagen IV, and perlecan. The MRL mouse, a mouse model for systemic lupus erythematosus, was serendipitously found to regenerate multiple ear punches [93]. Unlike other mice, the MRL mouse forms a basement membrane during wound repair that is then removed during ear punch regeneration; this was found to be correlated to increased MMP activity and decreased TIMP activity [94].

### 3.4. MMP Activity and Wound Healing

MMPs are a family of zinc-dependent proteases and have been associated with wound healing, which involves extensive remodeling of the ECM [95, 96]. Wound sites express many MMPs, which facilitate various processes such as the infiltration of inflammatory cells, migration of fibroblasts, and angiogenesis. Although there is some substrate redundancy among MMPs, the interstitial collagenases are unique in their ability to degrade stromal collagens (types I, II, and III). These collagenases include MMP-1, MMP-8, MMP-13, and MMP-14 (MMP-14 is a membrane-bound MMP) [97]. An experiment in* Drosophila* demonstrated that a secreted MMP was required for basement membrane remodeling during wound healing [98], suggesting that MMP-14 does not play a major role in wound healing. MMP-13 is synthesized by cells in cartilage and bone and preferentially degrades type II collagen found in cartilage. MMP-8 is expressed primarily in neutrophils. MMP-1 (in humans) is expressed by most cells and can readily degrade all stromal collagens, but mainly types I and III. Human MMP-1 does not have an exact mouse homolog. MMP-1a (McolA) has only a 58% amino acid homology with human MMP-1 (and 74% nucleotide homology) [99]. This is in contrast with MMP-13, which shares >90% sequence homology with the mouse model. Murine MMP-13, unlike human MMP-13, has a broad expression profile, which is why it has served as a surrogate for MMP-1 in murine models [99, 100]. MMP-13-deficient mice exhibit normal wound healing [101]. Presumably, the loss of MMP-13 can be compensated for by other members of the MMP family, such as MMP-2 and MMP-14. MMP-8 was found to be upregulated in MMP-13-deficient wounds compared to controls; however, excess MMP-8 prevents proper tissue repair, as mice overexpressing MMP-8 demonstrate impaired wound healing [102].

Blocking the activity of MMPs with broad, nonspecific inhibitors results in delayed wound healing [103] and impaired stem cell migration and differentiation [104]. Mice with a mutation in collagen I that renders it insensitive to cleavage by MMP-1 demonstrate impaired tissue remodeling and severely delayed tissue healing [105, 106]. Many MMP-deficient mutants, however, do not demonstrate abnormalities in wound healing, with the exception of MMP3-deficient mice, which have a wound contraction defect [101], and MMP-8-deficient mice, which exhibit increased inflammation [107]. Therefore, the question remains as to whether an essential MMP has been found or if their contributions are due to multiple, overlapping MMPs.* MMP-9* and* MMP-13* double knockout mouse demonstrates delayed tissue healing, which is reversed upon topical treatment with recombinant MMP-9 and MMP-13 [108]. MMP-9 knockout mice displayed impaired cutaneous wound healing accompanied by defects in keratinocyte migration and collagen fibrillogenesis [109]; however, a lack of MMP-9 enhances the rate of wound closure in injured corneas [110]. Contrary to other MMPs, which are expressed at the front of advancing epithelial sheets and stimulate cell migration, MMP-9 acts to inhibit the rate of wound closure by inhibiting the replication of cells in the migrating epithelial sheet. Similarly, anti-MMP-9 treatments reduced fibrosis in soleus muscle regeneration [111]. Thus, although MMPs are essential for tissue regeneration, the specific role of each MMP is highly complex.

## 4. Digit/Appendage Regeneration in Humans

Children under the age of 10–15 have been documented to regenerate the tips of their fingers if the amputation is treated conservatively [9, 10]. Regeneration has been documented to restore finger shape, fingerprint, function, and the sense of touch. There were some cases where bone regrowth was documented; however, lengthening of the digit could have occurred via the distal growth of granulation tissue [11, 112]. Treatment of amputated digits with a skin flap prevents regeneration both in amphibians and in mammals [10, 113]. Similar conservative treatment of adult fingertip amputations has resulted in wound healing with no reported lengthening of the fingertip [114]; however, there has been a report of limited bone regrowth following surgical removal of the diaphysis of the 3rd phalanx in an adult [115]. Adult fingertip healing (in individuals over 15 years old) with some documentation of bone regrowth was reported after treatment with a biological dressing based on chitin utilizing a “Hyphecan cap” (Hainan Kangda Marine Biomedical Corp., China) [116]; however, the amputated tip did not always grow to the full length. The Hyphecan occlusive dressing was also used in the treatment of other fingertip injuries [117]; however, the use of this material for the treatment of fingertip injuries outside of Hong Kong appears to be limited. A similar dressing was also used to treat burns in mice and was demonstrated to promote healing due to its modulation of TGF*β*1 levels [118]. The use of a silver sulphadiazine dressing in 19 patients (aged 16 to 64 years) for the treatment of 21 distal fingertip amputations was reported with good to excellent results; however, documentation of bone regrowth (if any) was not presented [119]. Although regeneration is generally limited to the third phalanx in humans, there was a report of a child who suffered a crushing amputation at the proximal interphalangeal joints of her ring and little fingers and regenerated a distal phalanx with vestigial nail without the middle phalanx in her ring finger, though her little finger remained a stump [120].

### 4.1. Mouse Model for Digit/Appendage Regrowth

The newt and salamander regeneration models are useful for understanding the regeneration of an entire limb; however, as model systems, these are far removed from mammalian regeneration. The mouse is an ideal model to study digit tip regeneration, as the process in mice is similar to human fingertip regeneration. Both digit tips are similarly comprised of bones, tendons, muscles, skin, nerves, and blood vessels. Regeneration of the digit tip requires all these tissues to regrow in their proper locations and orientations to restore functionality. Several mouse models have been utilized to study digit regeneration [16]; however, there are differences between mammalian and axolotl regeneration besides their intrinsic regenerative abilities. For example, salamander limb regeneration is dependent on the presence of nerves; however, a denervated mouse digit tip can still regenerate, albeit at a reduced rate [121]. A recent study found that combinations of FGF8 and BMP7 gene therapy in neural cells in the dorsal root ganglia (DRG) were delivered to the limbs through the long axons of axolotls, suggesting major neural inputs of FGF and BMP in regulating blastema cell proliferation as well as controlling organ regeneration ability [22]. Denervation appears to affect the regenerative ability of the tissue by abrogating FGF signaling. FGF-2 is normally present in regenerating tissue but is not detectable after denervation [42]. Regenerating amphibians always form an AEC, which functionally mimics the AER during development; however, studies of the apical epithelium of regenerating digit tips are very limited, and there appears to be no AEC that forms in nonregenerating amputations [49].

### 4.2. Amputation Location

**Figure 3:**
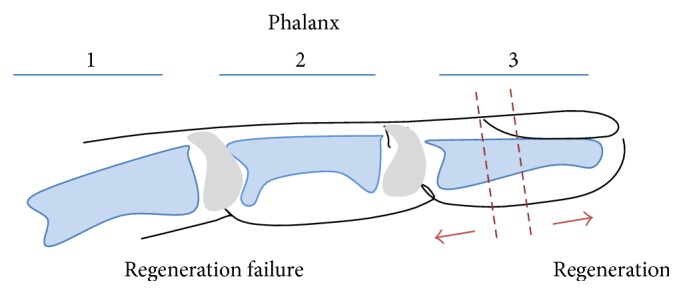
Amputation location affects the ability of digit/appendage to regenerate in mammals. Digit/appendage regrowth only occurs if the amputation site was distal to the middle to the 3rd phalanx, whereas digits amputated 2/3 through the 3rd phalanx do not regenerate.

Studies of digit tip regeneration in mice have indicated a sharp transition between a tip that will regenerate and one that will not [40, 42, 112]. Regeneration is limited to the middle of the third phalanx. An amputation proximal to this region will result in a nonregenerative response, as presented in Figure 3. The ability of the amputated fingertip to regenerate is thought to be correlated with the presence of the nail bed, which grows continuously throughout life. The germinal matrix of the nail bed contains adult stem cells which are thought to be involved in the regrowth of the amputated digit tip [42]. Additionally, bone regrowth has been correlated to nail regrowth, and there is no bone regrowth in distal amputations when the nail is surgically ablated. Conversely, there is bone regrowth in proximal amputations where the bone is removed from the ventral surface of the digit but not the nail and matrix [12]. A nail transplantation study in the amputated proximal phalanges of rats showed limited bone regrowth when the nail was transplanted; however, no bone regrowth was seen without nail transplantation [122]. Wnt pathway activation of the nail stem cells appears to be required in order for blastema growth and digit tip regeneration to occur [42, 43]; however, the relationship between the nail and regeneration of the terminal phalanx is still unclear, as there are case studies of regenerative failure even when the nail root was present. Therefore, it was hypothesized that the nail bed is necessary but not sufficient for successful regeneration, perhaps aiding the scarless healing process [112].

### 4.3. The MRL Mouse Model

The MRL/MpJ mouse strain has been commonly used as a model for autoimmune disease; it also has a unique capacity for wound healing and tissue regeneration without scar formation. Classically, this mouse strain displays an accelerated healing and tissue regeneration process after receiving an ear-hole punch. Moreover, 4-week-old MRL mice can regenerate their digits more quickly than control wild-type (WT) mice after having a distal digit amputated to the midpoint of the third phalanx [123]; however, when the digits from adult mice were amputated at the midpoint of the second phalanx, neither MRL mice nor controls could regenerate their digit tips [124, 125]. MRL mice (but not the WT controls) display blastema-like formation during the early stages after amputation; however, an apoptotic event eventually causes this structure to disappear. Altered ratios of collagens I and III, as well as differences in total collagen levels, have been demonstrated between MRL and WT mice, suggesting there would be differences in scar tissue formation, though not to the extent that there were differences in the regeneration process [125]. In a recent study, we showed that the prevention of fibrosis formation with MMP-1 therapy resulted in better soft tissue regeneration within the amputated digits of adult mice [126]. Thus, the deposition of collagen occurs through an essential balance between ECM reconstruction and tissue regeneration.

### 4.4. Stem Cells and Blastema/Nonblastemal Dedifferentiation

The regeneration of a newt or salamander limb is preceded by the formation of a proliferating blastema that is guided by the AEC. The mechanism of how this heterogeneous mass of dedifferentiated cells can then proceed to form a complete limb is still slowly being unraveled. Although there is no exact mammalian counterpart to the urodele blastema [127], digit tip regeneration in mice was shown to occur via the formation of a cluster of blastema-like mitotically active cells [128] that express BMP4 [112] as well as stem cell markers, including vimentin and Sca-1 [129]; however, the existence of a mammalian AEC during digit regeneration has not been demonstrated, which might explain the limited regeneration potential of the digit tip in mammals. Additionally, there is no evidence of dedifferentiation in the mammalian regenerating digit tip; however, this does not preclude the possibility that dedifferentiation may occur during mammalian digit tip regrowth.

Similar to lineage tracing studies in regenerating axolotl limbs and zebrafish fins, recent studies in mouse digit tip regeneration utilizing transgenic mice with Cre-mediated reporters corroborate the finding that the regenerated structures are lineage-dependent and derived from local tissues [40, 130]. Resident stem cells, which are already committed to become specific tissue types, are responsible for digit regeneration in mice; however, this does not rule out the possibility that terminally differentiated tissue can dedifferentiate into resident stem cells.

### 4.5. *Msx1*, Msx2, and BMP4

The* Msh homeobox (Msx) *type 1 and type 2 transcriptional repressors are both expressed near the nail bed of neonatal mice and at the tips of developing digits [6]. It has been suggested that* Msx-1* is required to maintain some cell types in an undifferentiated state and may be associated with urodele limb regeneration, and inactive msx genes also alter epithelial cell junction proteins during embryo implantation [131]. In amphibians,* Msx-1* is initially upregulated and then downregulated during regeneration [132]. Fetal mice deficient in* Msx-1,* but not* Msx-2*, do not readily regenerate amputated digit tips; however, this can be restored in culture in a dose-dependent manner with the addition of bone morphogenetic protein 4 (BMP4) [6]. This study also demonstrated that blocking BMP4 signaling using Noggin (a BMP inhibitor) prevented fetal digit regeneration. Hence, mammalian digit regeneration was shown to be dependent on* Msx1 *and BMP2 modulation [133].

## 5. Future Directions

### 5.1. Promotion of Dedifferentiation

Adult stem cells have been found to contribute to the regeneration of a number of human tissues which are present in bone marrow, intestinal mucosa, superficial layers of the skin, liver, and the nail bed; however, regeneration of complex structures such as digits or limbs requires a greater number of progenitor cells than that naturally present in adult tissues. Urodele amphibians overcome this deficiency by producing more progenitor cells via dedifferentiation of terminally differentiated cells in the blastema; hence, regeneration could be enhanced in mammals by increasing mammalian dedifferentiation. Dedifferentiation refers to the ability of terminally differentiated somatic cells to revert to a more plastic progenitor cell state. The methods of somatic cell nuclear transfer, chromosome transfer, or fusion with ES cells have all been used to induce totipotency or pluripotency [134, 135]. Utilizing a combination of transcription factors, fibroblast cells can be converted into induced pluripotent stem cells (iPS cells) [136]. C2C12 myotubes, which are mature differentiated multinucleated muscle cells, have been shown to dedifferentiate when induced to express* Msx-1* [137], the microtubule-binding molecule myoseverin [138], and the small molecule, reversine [139], or when treated with extracts from regenerating newt limbs [140]. Muscle cells that were dedifferentiated upon treatment with reversine could be redifferentiated under the appropriate lineage-specific inducing conditions into cells of different lineages, including osteoblasts and adipocytes. Another method of dedifferentiating myotubes is cell cycle reentry by means of inhibiting the tumor suppression genes Rb and ARF [76], which indicates that dedifferentiation may be possible in mammals. We were able to label terminally differentiated, multinucleated myotubes with *β*-galactosidase via a Cre-Lox system [141]. Following muscle injury, we observed *β*-galactosidase-positive mononuclear cells, which were able to differentiate into different types of muscle cells, suggesting that these progenitor cells were the result of mammalian dedifferentiation during wound healing.

### 5.2. Pathway Activation

A number of novel signaling pathways that are involved in cell proliferation and tissue growth have been revealed recently, such as the Wnt and Hippo pathways. In particular, Wnt pathway activation has been shown to be involved in digit regeneration [42, 43]; moreover, genes, including* LRP6 and LRP5*, that are related to Wnt/beta-catenin signal transduction have been found to be differentially expressed (higher expression) in MRL mice, which can form blastema-like structures, compared to DBA and C57BL mice [123].

The Hippo signaling pathway has also been shown to regulate cell proliferation and stem cell function. While its downstream effector Yes-Associated Protein (YAP) contributes to cancer development, its activation also has beneficial roles in regenerative medicine applications. In particular, the Hippo pathway has direct regulating effects on stem cell proliferation and maintenance [142, 143] which may be important for inducing the accumulation of blastema-like cells. Thus, developing molecular tools that can activate the Wnt or Hippo pathways in the amputated digits of mammals might be capable of enhancing the regeneration process [144].

### 5.3. Electrical Stimulation

The effect of electric fields on regeneration was revealed when newt limbs were induced to dedifferentiate by only applying an electric field (i.e., no amputation) strong enough to induce electroporation with the absence of cell necrosis or apoptosis. The time courses for changes in dedifferentiation and gene expression were similar to that occurring after amputation [145]. Quiescent, terminally differentiated cells are electrically polarized; however, tumor cells and stem cells are generally depolarized. The application of an electric field could represent a novel approach to promote digit/appendage regeneration and could be used in combination with other approaches (e.g., pathway activation or growth factor delivery).

## 6. Summary

Urodele amphibians such as newts and salamanders can regenerate large portions of their bodies, including an entire limb. Limb regeneration in mammals is much more limited with only a portion of the terminal phalanx being capable of regenerating, and this is generally further limited to neonatal or young mammals. Proximal amputation usually results in incomplete wound healing and scar formation. Studying the molecular mechanisms of amphibian regeneration and mammalian wound healing could lead to novel therapeutic strategies to augment the regenerative response beyond the current natural limits of regeneration in mammals, including humans.

## References

[1] Odelberg S. J. (2005). Cellular plasticity in vertebrate regeneration. *Anatomical Record Part B: New Anatomist*.

[2] Mescher A. L. (1996). The cellular basis of limb regeneration in urodeles. *International Journal of Developmental Biology*.

[3] Han M., Yang X., Taylor G., Burdsal C. A., Anderson R. A., Muneoka K. (2005). Limb regeneration in higher vertebrates: developing a roadmap. *Anatomical Record Part B: New Anatomist*.

[4] Borgens R. B. (1982). Mice regrow the tips of their foretoes. *Science*.

[5] Reginelli A. D., Wang Y. Q., Sassoon D., Muneoka K. (1995). Digit tip regeneration correlates with regions of Msx1 (Hox 7) expression in fetal and newborn mice. *Development*.

[6] Han M., Yang X., Farrington J. E., Muneoka K. (2003). Digit regeneration is regulated by Msx1 and BMP4 in fetal mice. *Development*.

[7] Neufeld D. A., Zhao W. (1993). Phalangeal regrowth in rodents: postamputational bone regrowth depends upon the level of amputation. *Progress in Clinical and Biological Research*.

[8] Masaki H., Ide H. (2007). Regeneration potency of mouse limbs. *Development Growth and Differentiation*.

[9] Douglas B. S. (1972). Conservative management of guillotine amputation of the finger in children. *Australian Paediatric Journal*.

[10] Illingworth C. M. (1974). Trapped fingers and amputated finger tips in children. *Journal of Pediatric Surgery*.

[11] Vidal P., Dickson M. G. (1993). Regeneration of the distal phalanx: a case report. *Journal of Hand Surgery*.

[12] Zhao W., Neufeld D. A. (1995). Bone regrowth in young mice stimulated by nail organ. *Journal of Experimental Zoology*.

[13] Mast B. A., Diegelmann R. F., Krummel T. M., Cohen I. K. (1992). Scarless wound healing in the mammalian fetus. *Surgery Gynecology and Obstetrics*.

[14] Samuels P., Tan A. K. W. (1999). Fetal scarless wound healing. *Journal of Otolaryngology*.

[15] Bullard K. M., Longaker M. T., Lorenz H. P. (2003). Fetal wound healing: current biology. *World Journal of Surgery*.

[16] Choi Y., Cox C., Lally K., Li Y. (2014). The strategy and method in modulating finger regeneration. *Regenerative Medicine*.

[17] Johnston A., Yuzwa S., Carr M. (2016). Dedifferentiated schwann cell precursors secreting paracrine factors are required for regeneration of the mammalian digit tip. *Cell Stem Cell*.

[18] Yu L., Yan M., Simkin J. (2014). Angiogenesis is inhibitory for mammalian digit regeneration. *Regeneration*.

[19] Sandoval-Guzmán T., Wang H., Khattak S. (2014). Fundamental differences in dedifferentiation and stem cell recruitment during skeletal muscle regeneration in two salamander species. *Cell Stem Cell*.

[20] Bryant S. V., Endo T., Gardiner D. M. (2002). Vertebrate limb regeneration and the origin of limb stem cells. *International Journal of Developmental Biology*.

[21] Suzuki M., Satoh A., Ide H., Tamura K. (2005). Nerve-dependent and -independent events in blastema formation during Xenopus froglet limb regeneration. *Developmental Biology*.

[22] Satoh A., Makanae A., Nishimoto Y., Mitogawa K. (2016). FGF and BMP derived from dorsal root ganglia regulate blastema induction in limb regeneration in *Ambystoma mexicanum*. *Developmental Biology*.

[23] Nogueira A. F., Costa C. M., Lorena J. (2016). Tetrapod limb and sarcopterygian fin regeneration share a core genetic programme. *Nature Communications*.

[24] Stocum D. L. (2011). The role of peripheral nerves in urodele limb regeneration. *European Journal of Neuroscience*.

[25] Stocum D. L., Cameron J. A. (2011). Looking proximally and distally: 100 years of limb regeneration and beyond. *Developmental Dynamics*.

[26] Crawford K., Stocum D. L. (1988). Retinoic acid coordinately proximalizes regenerate pattern and blastema differential affinity in axolotl limbs. *Development*.

[27] Iten L. E., Bryant S. V. (1975). The interaction between the blastema and stump in the establishment of the anterior-posterior and proximal-distal organization of the limb regenerate. *Developmental Biology*.

[28] Lo D. C., Allen F., Brockes J. P. (1993). Reversal of muscle differentiation during urodele limb regeneration. *Proceedings of the National Academy of Sciences of the United States of America*.

[29] Terada N., Hamazaki T., Oka M. (2002). Bone marrow cells adopt the phenotype of other cells by spontaneous cell fusion. *Nature*.

[30] Yang Z., Liu Q., Mannix R. J. (2014). Mononuclear cells from dedifferentiation of mouse myotubes display remarkable regenerative capability. *Stem Cells*.

[31] Kragl M., Knapp D., Nacu E. (2009). Cells keep a memory of their tissue origin during axolotl limb regeneration. *Nature*.

[32] Tu S., Johnson S. L. (2011). Fate restriction in the growing and regenerating zebrafish fin. *Developmental Cell*.

[33] Puri S., Folias A. E., Hebrok M. (2015). Plasticity and dedifferentiation within the pancreas: development, homeostasis, and disease. *Cell Stem Cell*.

[34] Tata P. R., Mou H., Pardo-Saganta A. (2013). Dedifferentiation of committed epithelial cells into stem cells in vivo. *Nature*.

[35] Kintner C. R., Brockes J. P. (1984). Monoclonal antibodies identify blastemal cells derived from dedifferentiating muscle in newt limb regeneration. *Nature*.

[36] Morrison J. I., Lööf S., He P., Simon A. (2006). Salamander limb regeneration involves the activation of a multipotent skeletal muscle satellite cell population. *Journal of Cell Biology*.

[37] Muneoka K., Fox W. F., Bryant S. V. (1986). Cellular contribution from dermis and cartilage to the regenerating limb blastema in axolotls. *Developmental Biology*.

[38] Gardiner D. M., Muneoka K., Bryant S. V. (1986). The migration of dermal cells during blastema formation in axolotls. *Developmental Biology*.

[39] Li C., Suttie J. M., Clark D. E. (2005). Histological examination of antler regeneration in red deer (*Cervus elaphus*). *The Anatomical Record. Part A, Discoveries in Molecular, Cellular, and Evolutionary Biology*.

[40] Rinkevich Y., Lindau P., Ueno H., Longaker M. T., Weissman I. L. (2011). Germ-layer and lineage-restricted stem/progenitors regenerate the mouse digit tip. *Nature*.

[41] Rinkevich Y., Maan Z. N., Walmsley G. G., Sen S. K. (2015). Injuries to appendage extremities and digit tips: a clinical and cellular update. *Developmental Dynamics*.

[42] Takeo M., Chou W. C., Sun Q. (2013). Wnt activation in nail epithelium couples nail growth to digit regeneration. *Nature*.

[43] Lehoczky J. A., Tabin C. J. (2015). Lgr6 marks nail stem cells and is required for digit tip regeneration. *Proceedings of the National Academy of Sciences of the United States of America*.

[44] Jopling C., Boue S., Belmonte J. C. I. (2011). Dedifferentiation, transdifferentiation and reprogramming: three routes to regeneration. *Nature Reviews Molecular Cell Biology*.

[45] Bhaskaran M., Kolliputi N., Wang Y., Gou D., Chintagari N. R., Liu L. (2007). Trans-differentiation of alveolar epithelial type II cells to type I cells involves autocrine signaling by transforming growth factor *β*1 through the Smad pathway. *The Journal of Biological Chemistry*.

[46] Pearton D. J., Yang Y., Dhouailly D. (2005). Transdifferentiation of corneal epithelium into epidermis occurs by means of a multistep process triggered by dermal development signals. *Proceedings of the National Academy of Sciences of the United States of America*.

[47] Boularaoui S. M., Abdel-Raouf K. M., Alwahab N. S. (2017). Efficient transdifferentiation of human dermal fibroblasts into skeletal muscle. *Journal of Tissue Engineering and Regenerative Medicine*.

[48] Phan A. Q., Lee J., Oei M. (2015). Positional information in axolotl and mouse limb extracellular matrix is mediated via heparan sulfate and fibroblast growth factor during limb regeneration in the axolotl (Ambystoma mexicanum). *Regeneration*.

[49] Satoh A., Bryant S. V., Gardiner D. M. (2012). Nerve signaling regulates basal keratinocyte proliferation in the blastema apical epithelial cap in the axolotl (Ambystoma mexicanum). *Developmental Biology*.

[50] Lévesque M., Gatien S., Finnson K. (2007). Transforming growth factor: *β* signaling is essential for limb regeneration in axolotls. *PLoS ONE*.

[51] Chablais F., Jaźwińska A. (2010). IGF signaling between blastema and wound epidermis is required for fin regeneration. *Development*.

[52] Zielins E. R., Ransom R. C., Leavitt T. E., Longaker M. T., Wan D. C. (2016). The role of stem cells in limb regeneration. *Organogenesis*.

[53] McCusker C., Bryant S. V., Gardiner D. M. (2015). The axolotl limb blastema: cellular and molecular mechanisms driving blastema formation and limb regeneration in tetrapods. *Regeneration*.

[54] Kumar A., Godwin J. W., Gates P. B., Garza-Garcia A. A., Brockes J. P. (2007). Molecular basis for the nerve dependence of limb regeneration in an adult vertebrate. *Science*.

[55] Boilly B., Cavanaugh K. P., Thomas D., Hondermarck H., Bryant S. V., Bradshaw R. A. (1991). Acidic fibroblast growth factor is present in regenerating limb blastemas of axolotls and binds specifically to blastema tissues. *Developmental Biology*.

[56] Munaim S. I., Mescher A. L. (1986). Transferrin and the trophic effect of neural tissue on amphibian limb regeneration blastemas. *Developmental Biology*.

[57] Wang L., Marchionni M. A., Tassava R. A. (2000). Cloning and neuronal expression of a type III newt neuregulin and rescue of denervated, nerve-dependent newt limb blastemas by rhGGF2. *Journal of Neurobiology*.

[58] Hay E. D., Fischman D. A. (1961). Origin of the blastema in regenerating limbs of the newt *Triturus viridescens*. An autoradiographic study using tritiated thymidine to follow cell proliferation and migration. *Developmental Biology*.

[59] Simkin J., Sammarco M. C., Dawson L. A., Schanes P. P., Yu L., Muneoka K. (2015). The mammalian blastema: regeneration at our fingertips. *Regeneration*.

[60] Tamura K., Ohgo S., Yokoyama H. (2010). Limb blastema cell: a stem cell for morphological regeneration. *Development Growth and Differentiation*.

[61] Kragl M., Tanaka E. M. (2009). Axolotl (Ambystoma mexicanum) limb and tail amputation. *Cold Spring Harbor Protocols*.

[62] Wu Y., Wang K., Karapetyan A. (2013). Connective tissue fibroblast properties are position-dependent during mouse digit tip regeneration. *PLoS ONE*.

[63] Zeller R., Jackson-Grusby L., Leder P. (1989). The limb deformity gene is required for apical ectodermal ridge differentiation and anteroposterior limb pattern formation. *Genes & Development*.

[64] Satoh A., Makanae A., Wada N. (2010). The apical ectodermal ridge (AER) can be re-induced by wounding, wnt-2b, and fgf-10 in the chicken limb bud. *Developmental Biology*.

[65] Zakany J., Zacchetti G., Duboule D. (2007). Interactions between HOXD and Gli3 genes control the limb apical ectodermal ridge via Fgf10. *Developmental Biology*.

[66] Godwin J. W., Pinto A. R., Rosenthal N. A. (2013). Macrophages are required for adult salamander limb regeneration. *Proceedings of the National Academy of Sciences of the United States of America*.

[67] Mercader N., Tanaka E. M., Torres M. (2005). Proximodistal identity during vertebrate limb regeneration is regulated by Meis homeodomain proteins. *Development*.

[68] Bhat T. A., Nambiar D., Tailor D., Pal A., Agarwal R., Singh R. P. (2013). Acacetin inhibits in vitro and in vivo angiogenesis and downregulates Stat signaling and VEGF expression. *Cancer Prevention Research*.

[69] Vinarsky V., Atkinson D. L., Stevenson T. J., Keating M. T., Odelberg S. J. (2005). Normal newt limb regeneration requires matrix metalloproteinase function. *Developmental Biology*.

[70] Santosh N., Windsor L. J., Mahmoudi B. S. (2011). Matrix metalloproteinase expression during blastema formation in regeneration-competent versus regeneration-deficient amphibian limbs. *Developmental Dynamics*.

[71] Stevenson T. J., Vinarsky V., Atkinson D. L., Keating M. T., Odelberg S. J. (2006). Tissue inhibitor of metalloproteinase 1 regulates matrix metalloproteinase activity during newt limb regeneration. *Developmental Dynamics*.

[72] Calve S., Odelberg S. J., Simon H.-G. (2010). A transitional extracellular matrix instructs cell behavior during muscle regeneration. *Developmental Biology*.

[73] Forsberg E., Hirsch E., Fröhlich L. (1996). Skin wounds and severed nerves heal normally in mice lacking tenascin-C. *Proceedings of the National Academy of Sciences of the United States of America*.

[74] Milner D. J., Cameron J. A. (2013). Muscle repair and regeneration: stem cells, scaffolds, and the contributions of skeletal muscle to amphibian limb regeneration. *Current Topics in Microbiology and Immunology*.

[75] Larson B. J., Longaker M. T., Lorenz H. P. (2010). Scarless fetal wound healing: a basic science review. *Plastic and Reconstructive Surgery*.

[76] Pajcini K. V., Corbel S. Y., Sage J., Pomerantz J. H., Blau H. M. (2010). Transient inactivation of Rb and ARF yields regenerative cells from postmitotic mammalian muscle. *Cell Stem Cell*.

[77] Levin M. (2009). Bioelectric mechanisms in regeneration: unique aspects and future perspectives. *Seminars in Cell and Developmental Biology*.

[78] Schilling J. A. (1976). Wound healing. *Surgical Clinics of North America*.

[79] Yokoyama H. (2008). Initiation of limb regeneration: the critical steps for regenerative capacity. *Development Growth and Differentiation*.

[80] Sammarco M. C., Simkin J., Cammack A. J. (2015). Hyperbaric oxygen promotes proximal bone regeneration and organized collagen composition during digit regeneration. *PLoS ONE*.

[81] Martin P., D'Souza D., Martin J. (2003). Wound healing in the PU.1 null mouse—tissue repair is not dependent on inflammatory cells. *Current Biology*.

[82] Gurtner G. C., Werner S., Barrandon Y., Longaker M. T. (2008). Wound repair and regeneration. *Nature*.

[83] Amendt C., Mann A., Schirmacher P., Blessing M. (2002). Resistance of keratinocytes to TGF*β*-mediated growth restriction and apoptosis induction accelerates re-epitheliazation in skin wounds. *Journal of Cell Science*.

[84] Ashcroft G. S., Yang X., Glick A. B. (1999). Mice lacking Smad3 show accelerated wound healing and an impaired local inflammatory response. *Nature Cell Biology*.

[85] Broughton G., Janis J. E., Attinger C. E. (2006). The basic science of wound healing. *Plastic and Reconstructive Surgery*.

[86] Li Y., Foster W., Deasy B. M. (2004). Transforming growth factor-*β*1 induces the differentiation of myogenic cells into fibrotic cells in injured skeletal muscle: a key event in muscle fibrogenesis. *American Journal of Pathology*.

[87] Witte M. B., Barbul A. (1997). General principles of wound healing. *Surgical Clinics of North America*.

[88] Lovvorn H. N., Cheung D. T., Nimni M. E., Perelman N., Estes J. M., Adzick N. S. (1999). Relative distribution and crosslinking of collagen distinguish fetal from adult sheep wound repair. *Journal of Pediatric Surgery*.

[89] Due E., Rossen K., Sorensen L. T., Kliem A., Karlsmark T., Haedersdal M. (2007). Effect of UV irradiation on cutaneous cicatrices: a randomized, controlled trial with clinical, skin reflectance, histological, immunohistochemical and biochemical evaluations. *Acta Dermato-Venereologica*.

[90] Globus M., Vethamany-Globus S., Lee Y. C. I. (1980). Effect of apical epidermal cap on mitotic cycle and cartilage differentiation in regeneration blastemata in the newt, Notophthalmus viridescens. *Developmental Biology*.

[91] Stocum D. L., Crawford K. (1987). Use of retinoids to analyze the cellular basis of positional memory in regenerating amphibian limbs. *Biochemistry and Cell Biology*.

[92] Baranowsky A., Mokkapati S., Bechtel M. (2010). Impaired wound healing in mice lacking the basement membrane protein nidogen 1. *Matrix Biology*.

[93] Clark L. D., Clark R. K., Heber-Katz E. (1998). A new murine model for mammalian wound repair and regeneration. *Clinical Immunology and Immunopathology*.

[94] Gourevitch D., Clark L., Chen P., Seitz A., Samulewicz S. J., Heber-Katz E. (2003). Matrix metalloproteinase activity correlates with blastema formation in the regenerating MRL mouse ear hole model. *Developmental Dynamics*.

[95] Parks W. C., Wilson C. L., López-Boado Y. S. (2004). Matrix metalloproteinases as modulators of inflammation and innate immunity. *Nature Reviews Immunology*.

[96] Bellayr I. H., Mu X., Li Y. (2009). Biochemical insights into the role of matrix metalloproteinases in regeneration: challenges and recent developments. *Future Medicinal Chemistry*.

[97] Coon C. I., Fiering S., Gaudet J., Wyatt C. A., Brinckerhoff C. E. (2009). Site controlled transgenic mice validating increased expression from human matrix metalloproteinase (MMP-1) promoter due to a naturally occurring SNP. *Matrix Biology*.

[98] Stevens L. J., Page-McCaw A. (2012). A secreted MMP is required for reepithelialization during wound healing. *Molecular Biology of the Cell*.

[99] Balbín M., Fueyo A., Knäuper V. (2001). Identification and enzymatic characterization of two diverging murine counterparts of human interstitial collagenase (MMP-1) expressed at sites of embryo implantation. *Journal of Biological Chemistry*.

[100] Brinckerhoff C. E., Matrisian L. M. (2002). Matrix metalloproteinases: a tail of a frog that became a prince. *Nature Reviews Molecular Cell Biology*.

[101] Hartenstein B., Dittrich B. T., Stickens D. (2006). Epidermal development and wound healing in matrix metalloproteinase 13-deficient mice. *Journal of Investigative Dermatology*.

[102] Danielsen P. L., Holst A. V., Maltesen H. R. (2011). Matrix metalloproteinase-8 overexpression prevents proper tissue repair. *Surgery*.

[103] Mirastschijski U., Haaksma C. J., Tomasek J. J., Ågren M. S. (2004). Matrix metalloproteinase inhibitor GM 6001 attenuates keratinocyte migration, contraction and myofibroblast formation in skin wounds. *Experimental Cell Research*.

[104] Bellayr I., Holden K., Mu X., Pan H., Li Y. (2013). Matrix metalloproteinase inhibition negatively affects muscle stem cell behavior. *International Journal of Clinical and Experimental Pathology*.

[105] Liu X., Wu H., Byrne M., Jeffrey J., Krane S., Jaenisch R. (1995). A targeted mutation at the known collagenase cleavage site in mouse type I collagen impairs tissue remodeling. *Journal of Cell Biology*.

[106] Beare A. H. M., O'Kane S., Krane S. M., Ferguson M. W. J. (2003). Severely impaired wound healing in the collagenase-resistant mouse. *Journal of Investigative Dermatology*.

[107] Gutiérrez-Fernández A., Inada M., Balbín M. (2007). Increased inflammation delays wound healing in mice deficient in collagenase-2 (MMP-8). *The FASEB Journal*.

[108] Hattori N., Mochizuki S., Kishi K. (2009). MMP-13 plays a role in keratinocyte migration, angiogenesis, and contraction in mouse skin wound healing. *American Journal of Pathology*.

[109] Kyriakides T. R., Wulsin D., Skokos E. A. (2009). Mice that lack matrix metalloproteinase-9 display delayed wound healing associated with delayed reepithelization and disordered collagen fibrillogenesis. *Matrix Biology*.

[110] Mohan R., Chintala S. K., Jung J. C. (2002). Matrix metalloproteinase gelatinase B (MMP-9) coordinates and effects epithelial regeneration. *Journal of Biological Chemistry*.

[111] Zimowska M., Olszynski K. H., Swierczynska M., Streminska W., Ciemerych M. A. (2012). Decrease of MMP-9 activity improves soleus muscle regeneration. *Tissue Engineering. Part A*.

[112] Han M., Yang X., Lee J., Allan C. H., Muneoka K. (2008). Development and regeneration of the neonatal digit tip in mice. *Developmental Biology*.

[113] Mescher A. L. (1976). Effects on adult newt limb regeneration of partial and complete skin flaps over the amputation surface. *Journal of Experimental Zoology*.

[114] Allen M. J. (1980). Conservative management of finger tip injuries in adults. *Hand*.

[115] McKim L. H. (1932). Regeneration of the distal phalanx. *Canadian Medical Association Journal*.

[116] Lee L. P., Lau P. Y., Chan C. W. (1995). A simple and efficient treatment for fingertip injuries. *Journal of Hand Surgery (British and European Volume)*.

[117] Halim A. S., Stone C. A., Devaraj V. S. (1998). The Hyphecan cap: a biological fingertip dressing. *Injury*.

[118] Baxter R. M., Dai T., Kimball J. (2013). Chitosan dressing promotes healing in third degree burns in mice: gene expression analysis shows biphasic effects for rapid tissue regeneration and decreased fibrotic signaling. *Journal of Biomedical Materials Research. Part A*.

[119] Buckley S. C., Scott S., Das K. (2000). Late review of the use of silver sulphadiazine dressings for the treatment of fingertip injuries. *Injury*.

[120] Cobiella C. E., Haddad F. S., Bacarese-Hamilton I. (1997). Phalangeal metaplasia following amputation in a child's finger. *Injury*.

[121] Mohammad K. S., Neufeld D. A. (2000). Denervation retards but does not prevent toetip regeneration. *Wound Repair and Regeneration*.

[122] Mohammad K. S., Day F. A., Neufeld D. A. (1999). Bone growth is induced by nail transplantation in amputated proximal phalanges. *Calcified Tissue International*.

[123] Chadwick R. B., Bu L., Yu H. (2007). Digit tip regrowth and differential gene expression in MRL/Mpj, DBA/2, and C57BL/6 mice. *Wound Repair and Regeneration*.

[124] Turner N. J., Johnson S. A., Badylak S. F. (2011). A histomorphologic study of the normal healing response following digit amputation in C57bl/6 and MRL/MpJ mice. *Archives of Histology and Cytology*.

[125] Gourevitch D. L., Clark L., Bedelbaeva K., Leferovich J., Heber-Katz E. (2009). Dynamic changes after murine digit amputation: the MRL mouse digit shows waves of tissue remodeling, growth, and apoptosis. *Wound Repair and Regeneration*.

[126] Mu X., Bellayr I., Pan H., Choi Y., Li Y. (2013). Regeneration of soft tissues is promoted by MMP1 treatment after digit amputation in mice. *PLoS ONE*.

[127] Muneoka K., Allan C. H., Yang X., Lee J., Han M. (2008). Mammalian regeneration and regenerative medicine. *Birth Defects Research Part C - Embryo Today: Reviews*.

[128] Neufeld D. A. (1980). Partial blastema formation after amputation in adult mice. *Journal of Experimental Zoology*.

[129] Fernando W. A., Leininger E., Simkin J. (2011). Wound healing and blastema formation in regenerating digit tips of adult mice. *Developmental Biology*.

[130] Lehoczky J. A., Robert B., Tabin C. J. (2011). Mouse digit tip regeneration is mediated by fate-restricted progenitor cells. *Proceedings of the National Academy of Sciences of the United States of America*.

[131] Sun X., Park C. B., Deng W., Potter S. S., Dey S. K. (2016). Uterine inactivation of muscle segment homeobox (Msx) genes alters epithelial cell junction proteins during embryo implantation. *FASEB Journal*.

[132] Crews L., Gates P. B., Brown R. (1995). Expression and activity of the newt Msx-1 gene in relation to limb regeneration. *Proceedings of the Royal Society B: Biological Sciences*.

[133] Yu L., Han M., Yan M., Lee E.-C., Lee J., Muneoka K. (2010). BMP signaling induces digit regeneration in neonatal mice. *Development*.

[134] Hochedlinger K., Jaenisch R. (2006). Nuclear reprogramming and pluripotency. *Nature*.

[135] Cowan C. A., Atienza J., Melton D. A., Eggan K. (2005). Developmental biology: nuclear reprogramming of somatic cells after fusion with human embryonic stem cells. *Science*.

[136] Takahashi K., Yamanaka S. (2006). Induction of pluripotent stem cells from mouse embryonic and adult fibroblast cultures by defined factors. *Cell*.

[137] Odelberg S. J., Kollhoff A., Keating M. T. (2000). Dedifferentiation of mammalian myotubes induced by msx1. *Cell*.

[138] Rosania G. R., Chang Y.-T., Perez O. (2000). Myoseverin, a microtubule-binding molecule with novel cellular effects. *Nature Biotechnology*.

[139] Chen S., Takanashi S., Zhang Q. (2007). Reversine increases the plasticity of lineage-committed mammalian cells. *Proceedings of the National Academy of Sciences of the United States of America*.

[140] McGann C. J., Odelberg S. J., Keating M. T. (2001). Mammalian myotube dedifferentiation induced by newt regeneration extract. *Proceedings of the National Academy of Sciences of the United States of America*.

[141] Mu X., Peng H., Pan H., Huard J., Li Y. (2011). Study of muscle cell dedifferentiation after skeletal muscle injury of mice with a Cre-Lox system. *PLoS ONE*.

[142] Vermeulen L. (2013). Keeping stem cells in check: a Hippo balancing act. *Cell Stem Cell*.

[143] Mullen A. C. (2014). Hippo tips the TGF-*β* scale in favor of pluripotency. *Cell Stem Cell*.

[144] Basu D., Lettan R., Damodaran K., Strellec S., Reyes-Mugica M., Rebbaa A. (2014). Identification, mechanism of action, and antitumor activity of a small molecule inhibitor of Hippo, TGF-*β*, and Wnt signaling pathways. *Molecular Cancer Therapeutics*.

[145] Atkinson D. L., Stevenson T. J., Park E. J., Riedy M. D., Milash B., Odelberg S. J. (2006). Cellular electroporation induces dedifferentiation in intact newt limbs. *Developmental Biology*.

